# New nonlinear model of population growth

**DOI:** 10.1371/journal.pone.0184728

**Published:** 2017-10-24

**Authors:** Badr Saad T. Alkahtani, Abdon Atangana, Ilknur Koca

**Affiliations:** 1 Department of Mathematical, Colle of Science, King Saud University, P.O.Box 1142, Riyadh, 11989, Saudi Arabia; 2 Institute for Groundwater Studies, Faculty of Natural and Agricultural Sciences University of the Free State, Bloemfontein 9300, South Africa; 3 Department of Mathematics, Faculty of Sciences, Mehmet Akif Ersoy University, 15100, Burdur, Turkey; Lanzhou University of Technology, CHINA

## Abstract

The model of population growth is revised in this paper. A new model is proposed based on the concept of fractional differentiation that uses the generalized Mittag-Leffler function as kernel of differentiation. The new model includes the choice of sexuality. The existence of unique solution is investigated and numerical solution is provided.

## Introduction

Researchers within the field of biology and mathematical biology are interested to know whether or not the certain specie will be instinct or not. This study has fascinated many researchers around the world in recent passed years. For instance to control the spread of a given infectious diseases researchers are interested in their reproductive number, that help to know whether or not the disease will be extinct [1-7]. However if the model is accurate enough they can give reliable predictions, if the predictions show the extinction of a given species, then laws-makers can take some decisions to protect the specie. We can find many examples of this in developed countries, for instance in South Africa, the government gave strict law against the killing of rhinos. In China, we have the protection of the tigers. A global protection of whale in all oceans and Africans elephants that are nowadays consider as rare species [4-7]. In case of infectious diseases, the aim is to end the spread of the virus that can considered as a specie, in this case also the control can be done via mathematical predictions. It is therefore important that in both cased the mathematical formulas should be able to portray more accurately the dynamic of the specie in time [8-9]. Generally speaking mathematical models allows a better thoughtful of how the complex interfaces and processes work. Indeed exhibiting of dynamic interactions in nature can provide a manageable way of understanding how numbers alter in excess of time or relation to each other. The aim of this paper is to provide a new model that will be able to describe the population growth more accurately.

## Model of population growth

Biological population demonstrating is worried with the changes in populace size and age spreading within a population as a significance of collaborations of creatures with the physical setting, with individuals of their own species, and with bacteria of other kinds. The biosphere is full of interfaces that varies from modest to dynamic. Earth’s processes affect human life and are momentously stochastic and seem disordered to naked eye. Nevertheless, a embarrassment of patterns can be perceived and are brought forth by using inhabitants demonstrating as an instrument. Population reproductions are employed to control maximum fruitage for agriculturists, to comprehend the changing aspects of biotic annexations, and have plentiful environmental safeguarding insinuations [8-9]. Thomas Malthus was one of the former to demonstrate that, inhabitants evolved with symmetrical configuration despite the fact that envisioning the providence of humankind [8-9]. Nonetheless, Nurgaliev’s law is a mathematical equation that portrays the rate of change of proportions of a population at a given time, in terms of the current population size. It is a deterministic conventional discrepancy equation in which the rate change is articulated as a quadratic function of the population size and this equation is given as:

$$\begin{array}{c} \frac{dn(t)}{dt}=an^{2}\left(t\right)-bn\left(t\right) \end{array}$$ (1)

In this equation, the size of a population is denoted by *n* time is measured in years, *a* is half of the average probability of a birth of male also for females, of a potential arbitrary parents pair within *a* is year. *b* is an average probability of a death of a person within a year. The above model has some limitations, the variation of growth of population is an average between two given times, which is not naturally true because the averaging is not the same at the different interval of time. The second problem is that the model does not take into account the choice of sex and also the memory effect. In this work we shall introduce new parameters to the model and also consider the memory effect induces by the fractional differentiation based on the Mittag-Leffler function.

## Fractional differentiation

The topic of fractional differentiation is one of the hot topics nowadays in almost all the fields of science, technology and engineering due to its wide applicability and also its ability of model real world problems more accurately than the classical differentiation. The first definition was proposed by Riemann and Liouville is given below as [10-14]

$$\begin{array}{c} {}^{RL}D_{t}^{\alpha }\left[f\left(t\right)\right]=\frac{1}{\Gamma (1-\alpha )}\frac{d}{dt}\int_{0}^{t}{\left(t-y\right)}^{-\alpha }f\left(y\right)dy,\;0<\alpha \leq 1. \end{array}$$ (2)

Caputo when working with a real world problem later modified this definition, as he was unable to recover the usual initial conditions, then Caputo modified Eq (2) by putting the derivative inside the integral to obtain:

$$\begin{array}{c} {}^{C}D_{t}^{\alpha }\left[f\left(t\right)\right]=\frac{1}{\Gamma (1-\alpha )}\int_{0}^{t}{\left(t-y\right)}^{-\alpha }\frac{d}{dy}f\left(y\right)dy,\;0<\alpha \leq 1. \end{array}$$ (3)

Definition (2) and (3) have been intensively used and misused in the last decades in many fields. However, when looking at the definition, we can see that, they are convolution of functions and the power law, or the kernel of transformation is the well-known power law. However, it is clear when looking at the behaviour of some physical phenomena that, not all of them follow the power law. Recently Caputo and Fabrizio suggested a step a head in fractional differentiation when they replaced the power law with exponential decay law as presented below [10-14]

$$\begin{array}{c} {}_{0}^{CF}D_{t}^{\alpha }\left[f\left(t\right)\right]=\frac{M(\alpha )}{1-\alpha }\int_{0}^{t}\frac{d}{dy}f\left(y\right)\exp\left[-\frac{\alpha }{1-\alpha }\left(t-y\right)\right]dy,\;0<\alpha <1. \end{array}$$ (4)

And Goufo and Atangana proposed the modified version in several research papers and it is given as follows [10-14]

$$\begin{array}{c} {}_{0}^{CFR}D_{t}^{\alpha }\left[f\left(t\right)\right]=\frac{M(\alpha )}{1-\alpha }\frac{d}{dt}\int_{0}^{t}f\left(y\right)\exp\left[-\frac{\alpha }{1-\alpha }\left(t-y\right)\right]dy,\;0<\alpha <1. \end{array}$$ (5)

But their proposition was rejected due to the criteria that need to be satisfied for an operator to be called fractional derivative. However their idea was great because a new kernel was introduced with no singularity. Atangana and Baleanu, to solve the problem in Caputo and Fabrizio operator, they suggested a new kernel based on the generalized Mittag-Leffler function, that is the more suitable function that was introduced to solve some problems of disc of convergence of power law. The function is also considered as the queen of fractional calculus and is more natural than power law. Their definitions are given below as follow [15-20]:

$$\begin{array}{c} {}_{0}^{ABC}D_{t}^{\alpha }\left[f\left(t\right)\right]=\frac{B(\alpha )}{1-\alpha }\int_{0}^{t}\frac{d}{dy}f\left(y\right)E_{\alpha }\left[-\frac{\alpha }{1-\alpha }{\left(t-y\right)}^{\alpha }\right]dy,\;0<\alpha <1, \end{array}$$ (6)

Also

$$\begin{array}{c} {}_{0}^{ABR}D_{t}^{\alpha }\left[f\left(t\right)\right]=\frac{B(\alpha )}{1-\alpha }\frac{d}{dt}\int_{0}^{t}f\left(y\right)E_{\alpha }\left[-\frac{\alpha }{1-\alpha }{\left(t-y\right)}^{\alpha }\right]dy,\;0<\alpha <1. \end{array}$$ (7)

This new version was applied in the theory of Chaos with great success therefore in this paper, we make use of this fractional differentiation to provide new model of population growth.

## New model of population growth

Many physical observed facts are said to follow the power law evolution. The use of fractional differentiation to predict the population growth was investigated before with Caputo power law fractional derivative in the following [20-23]. More importantly the expansion of mankind of population growth is obviously one of those one of those. However the chose of power law used to model such dynamical system must be chosen with care. The growth of population does take place exponentially as indicated by several classical models, or this does not take place with the trend of power law of *x*^*α*^ (the power law population growth can be observed in less developed countries were the rate of birth is very high). Additionally this does not occur with only a fading local memory (the real world situation for fading memory population can be found in developed countries where the rate of birth is very small as time goes on) as in the process of the diffusion within a porous media but rather combine both fading memory and power law. The only fractional operator that can with care and accurately replicate this dynamical system is perhaps the fractional differentiation with generalized Mittag-Leffler kernel. In this paper to accurately include into mathematical formulation the effect of fading memory and also power law, we convert the classical derivative with Atangana-Baleanu fractional derivative, which takes into account the power-law population growth together with fading local memory population growth. In this section, a new model of population growth is suggested using the concept of fractional, in addition to this, a new parameter taking into account the choice of partner will be introduced to well represent the physical investigation into mathematical formulas. Let assume *N*(*t*) to be the size of density population in a given period of time. Let *p* be the probability of an adult to choose a partner with same sex then the following equation is suggested:

$$\begin{array}{c} {}_{0}^{ABC}D_{t}^{\alpha }N\left(t\right)=aN^{2}\left(t\right)-bN\left(t\right)-\left(1-p\right)v\left(t\right)N^{2}\left(t\right) \end{array}$$ (8)

The new function *v*(*t*) is the selection function that a given individual will be convince to choose a partner with same sex. The new model induces also the memory effect due to the fractional differentiation.

We shall first present the equilibrium points of this dynamical system. To obtain them, we assume that the function is time independent therefore Eq (8) is transformed to

$$\begin{array}{ccc} aN^{2}-bN-\left(1-p\right)vN^{2} & = & 0\\ N & = & 0,\;N=\frac{b}{a-(1-p)v}\\ a & \neq & (1-p)v \end{array}$$ (9)

Therefore the realistic equilibrium point is when the proportion of the rate of death with the difference between the birth contribution and the factor of choice of partner. However if the following inequality holds *a* − (1 − *p*)*v* < 0 then mankind specie will die out. If *a* = (1 − *p*)*v* then in a near future also mankind will vanish. If the quantity is big enough then mankind will survive.

### Existence of solution

The conditions within which the new equation admits a positive solution will be discussed in this section. To do this, we consider *X* = *C*[*a*, *b*] the Banach space of every continuous real functions defined in the closed set [*a*, *b*], which bestowed with the sub norm and *Z* be the shaft defined as: *Z* = {*N* ∈ *X*, *N*(*t*) ≥ 0, *a* ≤ *t* ≤ *b*}. We shall present the following Banach fixed-point theorem that will be used for the existence demonstration.

**Definition 1:** Let *E* be a real Banach space with a cone *H*. *H* initiates a restricted order ≤ in *E* in the succeeding approach [18]

$$\begin{array}{c} x\leq y\Rightarrow y-x\in H. \end{array}$$

For every *x*, *y* ∈ *E* the order interval is defined as 〈*a*, *b*〉 = {*f* ∈ *E*: *a* ≤ *f* ≤ *b*}. *A* cone *K* is denoted normal, if one can find a positive constant *j* such that *h*, *d* ∈ *K*, *Φ* < *h* < *d* ⇒ ‖*h*‖ ≤ *j*‖*d*‖, where *Φ* denotes the zero element of *K*.

**Theorem 1** [18]: Let *H* be a closed set subspace of a Banach space of *D*. let *G* be a contraction mapping with Lipschitz constant *g* < 1 from *H* to *H*. Thus *G* possesses a fixed-point t* in *H*. In addition, if *t*_0_ is a random point in *H* and {*t*_*n*_} is a sequence defined by *t*_*n*+1_ = *Gt*_*n*_(*n* = 0, 1, 2…), then for a large number *n*, *t*_*n*_ tends to t* in *H* and $d\left(t_{n},t^{*}\right)\leq \frac{g^{n}}{\left(1-g\right)}d\left(t_{1},t_{0}\right)$.

We present also some properties of Atangana-Baleanu derivative in Caputo sense.

**Theorem 2** [19]: Let *f*(*t*)∈*H*^1^(*a*, *b*), *b* > *a* such that the Atangana-Baleanu fractional derivative exists, then the following relationship holds:

$$\begin{array}{c} {}_{0}^{AB}I_{t}^{\alpha }\left\{{}_{0}^{ABR}D_{t}^{\alpha }f\left(t\right)\right\}=f\left(t\right) \end{array}$$ (10)

$$\begin{array}{c} {}_{0}^{AB}I_{t}^{\alpha }\left\{{}_{0}^{ABC}D_{t}^{\alpha }f\left(t\right)\right\}=f\left(t\right)-f\left(0\right) \end{array}$$ (11)

**Proof**: By definition we establish the above relation (10) using the Laplace transform operator as follow:

$$\begin{array}{c} {}_{0}^{AB}I_{t}^{\alpha }\left\{{}_{0}^{ABR}D_{t}^{\alpha }f\left(t\right)\right\}=\frac{1-\alpha }{B(\alpha )}{\;}_{0}^{ABR}D_{t}^{\alpha }f\left(t\right)+\frac{\alpha }{B(\alpha )\Gamma (\alpha )}\int_{0}^{t}{\left(t-y\right)}^{\alpha -1\;}{}_{0}^{ABR}D_{y}^{\alpha }f\left(y\right)dy. \end{array}$$ (12)

Applying on both side of Eq (12) the Laplace transform, we obtain the following expression

$$\begin{array}{ccc} L\{{}_{0}^{AB}I_{t}^{\alpha }\left\{{}_{0}^{ABR}D_{t}^{\alpha }f\left(t\right)\right\}\} & = & \frac{1-\alpha }{B(\alpha )}L{\{}_{0}^{ABR}D_{t}^{\alpha }f\left(t\right)\}\\ & & +L\{\frac{\alpha }{B(\alpha )\Gamma (\alpha )}\int_{0}^{t}{\left(t-y\right)}^{\alpha -1\;}{}_{0}^{ABR}D_{y}^{\alpha }f\left(y\right)dy\} \end{array}$$ (13)

$$\begin{array}{ccc} L\{{}_{0}^{AB}I_{t}^{\alpha }\left\{{}_{0}^{ABR}D_{t}^{\alpha }f\left(t\right)\right\}\} & = & \frac{1-\alpha }{B(\alpha )}\frac{B(\alpha )}{1-\alpha }\frac{s^{\alpha }F\left(s\right)}{s^{\alpha }+\frac{\alpha }{1-\alpha }}\\ & & +\frac{\alpha }{B(\alpha )}\frac{B(\alpha )}{1-\alpha }s^{-\alpha }\frac{s^{\alpha }F\left(s\right)}{s^{\alpha }+\frac{\alpha }{1-\alpha }} \end{array}$$

$$\begin{array}{ccc} L\{{}_{0}^{AB}I_{t}^{\alpha }\left\{{}_{0}^{ABR}D_{t}^{\alpha }f\left(t\right)\right\}\} & = & \frac{s^{\alpha }F\left(s\right)}{s^{\alpha }+\frac{\alpha }{1-\alpha }}+\frac{\alpha }{1-\alpha }\frac{F(s)}{s^{\alpha }+\frac{\alpha }{1-\alpha }}\\ L\{{}_{0}^{AB}I_{t}^{\alpha }\left\{{}_{0}^{ABR}D_{t}^{\alpha }f\left(t\right)\right\}\} & = & F(s) \end{array}$$

By the inverse Laplace transform we obtain

$$\begin{array}{c} {}_{0}^{AB}I_{t}^{\alpha }\left\{{}_{0}^{ABR}D_{t}^{\alpha }f\left(t\right)\right\}=f\left(t\right) \end{array}$$ (14)

The prove Eq (10b) we use another method that consists of solving the following time fractional ordinary differential equation with Atangana-Baleanu derivative in Caputo sense

$$\begin{array}{c} {}_{0}^{ABC}D_{t}^{\alpha }f\left(t\right)=u\left(t\right) \end{array}$$ (15)

With the aim to find the function *f*(*t*), to do this we employ again the Laplace transform on both sides we obtain

$$\begin{array}{c} \frac{B(\alpha )}{1-\alpha }\frac{s^{\alpha }F\left(s\right)-s^{\alpha -1}f\left(0\right)}{s^{\alpha }+\frac{\alpha }{1-\alpha }}=U\left(s\right) \end{array}$$ (16)

Rearranging we obtain

$$\begin{array}{ccc} F(s) & = & \frac{1-\alpha }{B(\alpha )}\frac{\left(s^{\alpha }+\frac{\alpha }{1-\alpha }\right)}{s^{\alpha }}U\left(s\right)+s^{-1}f\left(0\right)\\ f(t) & = & \frac{1-\alpha }{B(\alpha )}u\left(t\right)+\frac{\alpha }{B(\alpha )\Gamma (\alpha )}\int_{0}^{t}u\left(y\right){\left(t-y\right)}^{\alpha -1}dy+f\left(0\right)\\ f(t)-f(0) & = & \frac{\alpha }{B(\alpha )\Gamma (\alpha )}\int_{0}^{t}u\left(y\right){\left(t-y\right)}^{\alpha -1}dy+\frac{1-\alpha }{B(\alpha )}u\left(t\right)\\ f(t)-f(0) & = & {}_{0}^{AB}I_{t}^{\alpha }u\left(t\right) \end{array}$$ (17)

This completes the proof.

Here applying the AB-fractional integral on Eq (8), we obtain the following

$$\begin{array}{ccc} N(t)-N(0) & = & \frac{1-\alpha }{AB(\alpha )}\left\{aN^{2}\left(t\right)-bN\left(t\right)-\left(1-p\right)v\left(t\right)N^{2}\left(t\right)\right\}\\ & & +\frac{\alpha }{AB(\alpha )\Gamma (\alpha )}\int_{0}^{t}{\left(t-y\right)}^{\alpha -1}\left\{\begin{array}{c} aN^{2}\left(y\right)-bN\left(y\right)\\ -\left(1-p\right)v\left(y\right)N^{2}\left(y\right) \end{array}\right\}dy \end{array}$$ (18)

It is important to note that, Eq (17) is equivalent to Eq (8), in this work, we will use Eq (17) to show the existence of Eq (8).

**Lemma 1**: The mapping *G*: *H* → *H* defined as

$$\begin{array}{ccc} GN(t) & = & \frac{1-\alpha }{AB(\alpha )}V\left(t,N\left(t\right)\right)\\ & & +\frac{\alpha }{AB(\alpha )\Gamma (\alpha )}\int_{0}^{t}{\left(t-y\right)}^{\alpha -1}V\left(y,N\left(y\right)\right)dy\\ V(t,N(t)) & = & aN^{2}\left(t\right)-bN\left(t\right)-\left(1-p\right)v\left(t\right)N^{2}\left(t\right) \end{array}$$ (19)

**Lemma 2**: Let *M* ⊂ *H* be bounded implying, we can find *l* > 0 such that,

$$\begin{array}{c} ∥N(a)-N(b)∥\leq l(a-b),\;\forall N\in M. \end{array}$$

Then $\bar{G(M)}$ is compact.

**Proof**: Let $I=\max\{\frac{1-\alpha }{AB(\alpha )}+V\left(t,N\left(t\right)\right\},$ 0 ≤ *N* ≤ *L*. For *N* ∈ *M*, we have the following

$$\begin{array}{ccc} ∥GN(t)∥ & \leq & \frac{1-\alpha }{AB(\alpha )}∥V\left(t,N\left(t\right)\right)∥\\ & & +\frac{\alpha }{AB(\alpha )\Gamma (\alpha )}\int_{0}^{t}{\left(t-y\right)}^{\alpha -1}∥V\left(y,N\left(y\right)\right)∥dy\\ & \leq & \frac{1-\alpha }{AB(\alpha )}I+\frac{\alpha }{AB(\alpha )}I\;\frac{t^{\alpha }}{\Gamma (\alpha +1)} \end{array}$$ (20)

This implies the function *G* is bounded.

Let us now consider *N* ∈ *M*, *t*_1_, *t*_2_, *t*_1_ < *t*_2_, then for a given *ϵ* > 0, if |*t*_2_ − *t*_1_| < Λ.

Then

$$\begin{array}{ccc} ∥GN\left(t_{2}\right)-GN\left(t_{1}\right)∥ & \leq & \frac{1-\alpha }{AB(\alpha )}∥V\left(t_{2},N\left(t_{2}\right)\right)-V\left(t_{1},N\left(t_{1}\right)\right)∥\\ & & +∥\begin{array}{c} \frac{\alpha }{AB(\alpha )\Gamma (\alpha )}\int_{0}^{t_{2}}{\left(t_{2}-y\right)}^{\alpha -1}∥V\left(y,N\left(y\right)\right)∥dy\\ -\frac{\alpha }{AB(\alpha )\Gamma (\alpha )}\int_{0}^{t_{1}}{\left(t_{1}-y\right)}^{\alpha -1}∥V\left(y,N\left(y\right)\right)∥dy \end{array}∥\\ & \leq & \frac{1-\alpha }{AB(\alpha )}∥V\left(t_{2},N\left(t_{2}\right)\right)-V\left(t_{1},N\left(t_{1}\right)\right)∥\\ & & +\frac{\alpha L}{AB(\alpha )\Gamma (\alpha )}\left\{\int_{0}^{t_{2}}{\left(t_{2}-y\right)}^{\alpha -1}dy-\int_{0}^{t_{1}}{\left(t_{1}-y\right)}^{\alpha -1}dy\right\} \end{array}$$ (21)

We will treat the above inequality piece by piece we first start with the integral part.

$$\begin{array}{ccc} & & \int_{0}^{t_{2}}{\left(t_{2}-y\right)}^{\alpha -1}dy-\int_{0}^{t_{1}}{\left(t_{1}-y\right)}^{\alpha -1}dy\\ & = & \int_{0}^{t_{1}}\left\{{\left(t_{1}-y\right)}^{\alpha -1}-{\left(t_{2}-y\right)}^{\alpha -1}\right\}dy\\ +\int_{t_{1}}^{t_{2}}{\left(t_{2}-y\right)}^{\alpha -1}dy & = & \frac{2}{\Gamma (\alpha +1)}{\left(t_{2}-t_{1}\right)}^{\alpha } \end{array}$$ (22)

We next investigate the following

$$\begin{array}{ccc} & & ∥V\left(t_{2},N\left(t_{2}\right)\right)-V\left(t_{1},N\left(t_{1}\right)\right)∥\\ & = & ∥\begin{array}{c} a\left(N^{2}\left(t_{2}\right)-N^{2}\left(t_{1}\right)\right)-b\left(N\left(t_{2}\right)-N\left(t_{1}\right)\right)\\ -\left(1-p\right)(v\left(t_{2}\right)N^{2}\left(t_{2}\right)-v\left(t_{1}\right)N^{2}\left(t_{1}\right)) \end{array}∥\\ & \leq & |a|∥N^{2}\left(t_{2}\right)-N^{2}\left(t_{1}\right)∥+|b|∥N\left(t_{2}\right)-N\left(t_{1}\right)∥\\ & & +\left(1-p\right)∥N^{2}\left(t_{2}\right)-N^{2}\left(t_{1}\right)∥\\ & \leq & \left\{2aL+b+2L\left(1-p\right)\right\}∥N\left(t_{2}\right)-N\left(t_{1}\right)∥\\ & \leq & \left\{2aL+b+2L\left(1-p\right)\right\}l∥\left(t_{2}-t_{1}\right)∥\\ & \leq & J∥(t_{2}-t_{1})∥ \end{array}$$ (23)

Now putting Eqs (22) and (21) into (20) we obtain:

$$\begin{array}{ccc} ∥GN\left(t_{2}\right)-GN\left(t_{1}\right)∥ & \leq & \\ & & \frac{1-\alpha }{AB(\alpha )}J∥\left(t_{2}-t_{1}\right)∥+\frac{2\alpha }{AB(\alpha )\Gamma (\alpha +1)}{∥(t_{2}-t_{1})∥}^{\alpha } \end{array}$$ (24)

Therefore for each *ϵ* > 0, we can find

$$\begin{array}{c} \Lambda =\frac{\varepsilon }{\frac{1-\alpha }{AB(\alpha )}\left\{\left\{2aL+b+2L\left(1-p\right)\right\}l\right\}+\frac{2\alpha }{AB(\alpha )\Gamma (\alpha +1)}} \end{array}$$ (25)

Such that

$$\begin{array}{c} ∥GN\left(t_{2}\right)-GN\left(t_{1}\right)∥\leq \varepsilon \end{array}$$

Hencefort *G*(*M*) is equi-continuous and according to the well-known Arzela-Ascoli theorem, $\bar{G(M)}$ is compact.

**Theorem 3:** *V*: [*a*, *b*] × [0, ∞)→[0, ∞) be a continuous function and *V*(*t*,.) increasing for each *t* in [*a*, *b*]. Let us assume that one can find *v*, *w* satisfying *K*(*D*)*v* ≤ *V*(*t*, *v*), *K*(*D*)*w* ≥ *V*(*t*, *w*), 0 ≤ *v*(*t*)≤*w*(*t*), *a* ≤ *t* ≤ *b*. Then our new equation has a positive solution.

**Proof**: The fixed-point of the operator G is needed to be considered. Nevertheless, within the framework of lemma 1, the considered operator *G*: *H* → *H* is completely continuous. Let us choose two arbitrary densities of population in the *N*_1_ and *N*_2_ in *H* satisfying *N*_1_ ≤ *N*_2_ then, by assuming that, *V* is a positive function, then

$$\begin{array}{ccc} GN_{1}\left(t\right) & \leq & \frac{1-\alpha }{AB(\alpha )}∥V\left(t,N_{1}\left(t\right)\right)∥\\ & & +\frac{\alpha }{AB(\alpha )\Gamma (\alpha )}\int_{0}^{t}{\left(t-y\right)}^{\alpha -1}∥V\left(y,N_{1}\left(y\right)\right)∥dy\\ & \leq & GN_{2}\left(t\right) \end{array}$$ (26)

Henceforth the mapping *G* is increasing. By the conjecture, we get *Gm* ≥ *m*, *Gn* ≤ *n*. Henceforth the operator *G*: 〈*n*, *m*〉 → 〈*n*, *m*〉 is compact within the framework of lemma 2 and continuous in view of lemma 1. Since *H* is a normal cone of *G*.

### Uniqueness of solution

In this section, we discuss with care the conditions under which the unicity of the solution are obtained. To establish these conditions, we consider evaluating the following quantity.

$$\begin{array}{ccc} & & ∥GN(t)-GM(t)∥\\ & \leq & ∥\begin{array}{c} \frac{1-\alpha }{AB(\alpha )}\left(V\left(t,N\left(t\right)\right)-V\left(t,M\left(t\right)\right)\right)\\ +\frac{\alpha }{AB(\alpha )\Gamma (\alpha )}\int_{0}^{t}{\left(t-y\right)}^{\alpha -1}\left(V\left(y,N\left(y\right)\right)-V\left(y,M\left(y\right)\right)\right)dy \end{array}∥\\ & \leq & \frac{1-\alpha }{AB(\alpha )}∥V\left(t,N\left(t\right)\right)-V\left(t,M\left(t\right)\right)∥+\\ & & \frac{\alpha }{AB(\alpha )\Gamma (\alpha )}\int_{0}^{t}{\left(t-y\right)}^{\alpha -1}∥V\left(y,N\left(y\right)\right)-V\left(y,M\left(y\right)\right)∥dy\\ & \leq & \frac{1-\alpha }{AB(\alpha )}J∥N\left(t\right)-M\left(t\right)∥+\\ & & \frac{\alpha }{AB(\alpha )\Gamma (\alpha )}J\int_{0}^{t}{\left(t-y\right)}^{\alpha -1}∥N\left(t\right)-M\left(t\right)∥dy\\ ∥GN(t)-GM(t)∥ & \leq & \left\{\frac{1-\alpha }{AB(\alpha )}J+\frac{\alpha b^{\alpha }}{AB(\alpha )\Gamma (\alpha +1)}J\right\}∥N\left(t\right)-M\left(t\right)∥ \end{array}$$ (27)

Therefore if the following condition holds $\frac{1-\alpha }{AB(\alpha )}J+\frac{\alpha b^{\alpha }}{AB(\alpha )\Gamma (\alpha +1)}J<1$ then, the mapping *G* is a contraction, which implies it has a fixed-point, thus, the new model admits a unique positive solution.

## Numerical solution via forward-corrector method

The recent development of fractional differentiation based on the Mittag-Leffler function has induced a new type of Volterra fractional differential equations. As presented earlier, the fractional integral calculus associated o the new fractional calculus is the set of functions for which the their fractional integral in Atangana and Baleanu sense is an average of the given function and the Riemann-Liouville fractional integral. This new design has therefore opened way to many new studies, for instance what can we do to solve the Volterra version of a given equation numerically. It is well known that the Corrector method is very accurate method to handle Volterra equations, due to the Riemann-Liouville, however, with the new fractional integral one could possible apply also the corrector method in the integral part and apply another numerical method in the other part. In this section, we introduce the Forward-Corrector method to solve our new model. Nevertheless there are two ways to handle numerically fractional differential equation based on the new fractional differentiation. In our case, we could solve our problem directly in its present form or solve its Volterra version. We shall start with the Volterra version.

$$\begin{array}{ccc} N(t)-N(0) & = & \frac{1-\alpha }{AB(\alpha )}\left\{aN^{2}\left(t\right)-bN\left(t\right)-\left(1-p\right)v\left(t\right)N^{2}\left(t\right)\right\}\\ & & +\frac{\alpha }{AB(\alpha )\Gamma (\alpha )}\int_{0}^{t}{\left(t-y\right)}^{\alpha -1}\left\{\begin{array}{c} aN^{2}\left(y\right)-bN\left(y\right)\\ -\left(1-p\right)v\left(y\right)N^{2}\left(y\right) \end{array}\right\}dy \end{array}$$

The part within the integral could be handled with the Corrector method, which is provided as follow

$$\begin{array}{ccc} & & \int_{0}^{t_{n}}{\left(t_{n+1}-y\right)}^{\alpha -1}V\left(y,N\left(y\right)\right)dy\\ & = & \frac{h^{\alpha }}{\alpha (\alpha +1)}\sum_{j=0}^{n+1}b_{j,n+1}V\left(t_{j},N\left(t_{j}\right)\right)\\ b_{j,n+1} & = & \left\{ \begin{array}{cc} n^{\alpha +1}-\left(n-\alpha \right){\left(n+1\right)}^{\alpha }, & \;\text{if}\;j=0\\ {\left(n-j+2\right)}^{\alpha +1}-{\left(n-j\right)}^{\alpha +1}-2{\left(n-j+1\right)}^{\alpha +1} & \;\text{if}\;1\leq j\leq n,\\ 1, & \;\text{if}\;j=n+1 \end{array}\right. \end{array}$$ (28)

Therefore according to [18] the fractional variant of the one step Adam-Moulton method for the second part of our equation is given by:

$$\begin{array}{c} \frac{\alpha h^{\alpha }}{AB(\alpha )\Gamma (\alpha +2)}V\left(t_{n+1},N_{h}^{p}\left(t_{n+1}\right)\right)+\frac{\alpha h^{\alpha }}{AB(\alpha )\Gamma (\alpha +2)}\sum_{j=0}^{n}b_{j,n+1}V\left(t_{j},N_{h}\left(t_{j}\right)\right) \end{array}$$ (29)

In the second part we use the forward approximation as follows

$$\begin{array}{c} N\left(t_{n+1}\right)=N\left(t_{n}\right)+\frac{1-\alpha }{AB(\alpha )}hV\left(t_{n+1},N\left(t_{n+1}\right)\right) \end{array}$$ (30)

Putting Eqs (29) and (28) into Eq (17), we obtain the following numerical approximation:

$$\begin{array}{ccc} N(t_{n+1}) & = & N\left(t_{n}\right)+\frac{1-\alpha }{AB(\alpha )}hV\left(t_{n+1},N\left(t_{n+1}\right)\right)\\ & & \frac{\alpha h^{\alpha }}{AB(\alpha )\Gamma (\alpha +2)}V\left(t_{n+1},N_{h}^{p}\left(t_{n+1}\right)\right)\\ & & +\frac{\alpha h^{\alpha }}{AB(\alpha )\Gamma (\alpha +2)}\sum_{j=0}^{n}b_{j,n+1}V\left(t_{j},N_{h}\left(t_{j}\right)\right) \end{array}$$ (31)

This approach can be used to solve many other fractional differential equations based on the new fractional differentiation.

The second approach to solve our problem is to discretize the Atangana-Baleanu time fractional derivative. Koca and Atangana suggested the numerical approximation of the new derivative as follow [19]:

$$\begin{array}{ccc} & & {}_{0}^{ABC}D_{t}^{\alpha }\left(N\left(t_{n+1}\right)\right)\\ & = & \frac{AB(\alpha )}{1-\alpha }\sum_{k=1}^{n+1}\frac{N^{k+1}-N^{k}}{\Delta t}\left\{\begin{array}{c} \left(t_{n}-t_{k+1}\right)E_{\alpha ,2}\left(-\frac{\alpha }{1-\alpha }\left(t_{n}-t_{k+1}\right)\right)\\ -\left(t_{n}-t_{k}\right)E_{\alpha ,2}\left(-\frac{\alpha }{1-\alpha }\left(t_{n}-t_{k}\right)\right) \end{array}\right\} \end{array}$$ (32)

$$\begin{array}{c} E_{\alpha ,2}\left(z\right)=\sum_{j=0}^{\infty }\frac{z^{j}}{j!\Gamma (\alpha j+2)} \end{array}$$

Replacing the above in Eq (8), using also the forward numerical scheme, then the numerical approximation solution of the new model is given as:

$$\begin{array}{ccc} & & \frac{AB(\alpha )}{1-\alpha }\sum_{k=1}^{n+1}\frac{N^{k+1}-N^{k}}{\Delta t}\left\{\begin{array}{c} \left(t_{n}-t_{k+1}\right)E_{\alpha ,2}\left(-\frac{\alpha }{1-\alpha }\left(t_{n}-t_{k+1}\right)\right)\\ -\left(t_{n}-t_{k}\right)E_{\alpha ,2}\left(-\frac{\alpha }{1-\alpha }\left(t_{n}-t_{k}\right)\right) \end{array}\right\}\\ & & \;=aN^{2}\left(t_{n+1}\right)-bN\left(t_{n}\right)-\left(1-p\right)v\left(t_{n}\right)N^{2}\left(t_{n+1}\right)\\ & & \;\frac{AB(\alpha )}{1-\alpha }\sum_{k=1}^{n+1}\frac{N^{k+1}-N^{k}}{\Delta t}\beta_{k,n}\\ & & \;=aN^{2}\left(t_{n+1}\right)-bN\left(t_{n}\right)-\left(1-p\right)v\left(t_{n}\right)N^{2}\left(t_{n+1}\right) \end{array}$$ (33)

## Numerical simulations

**Fig 1:**
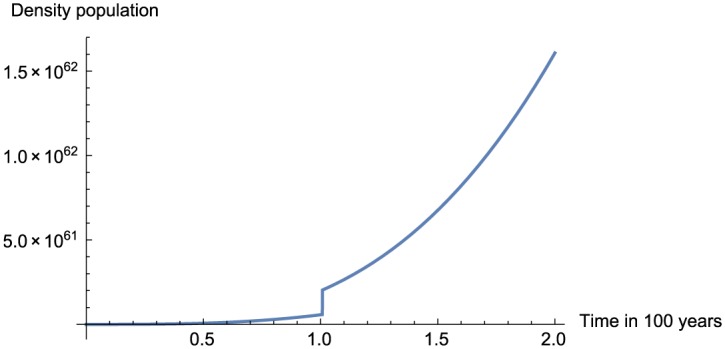
Numerical simulation for *α* = 0.95 and *t* = 100.

**Fig 2:**
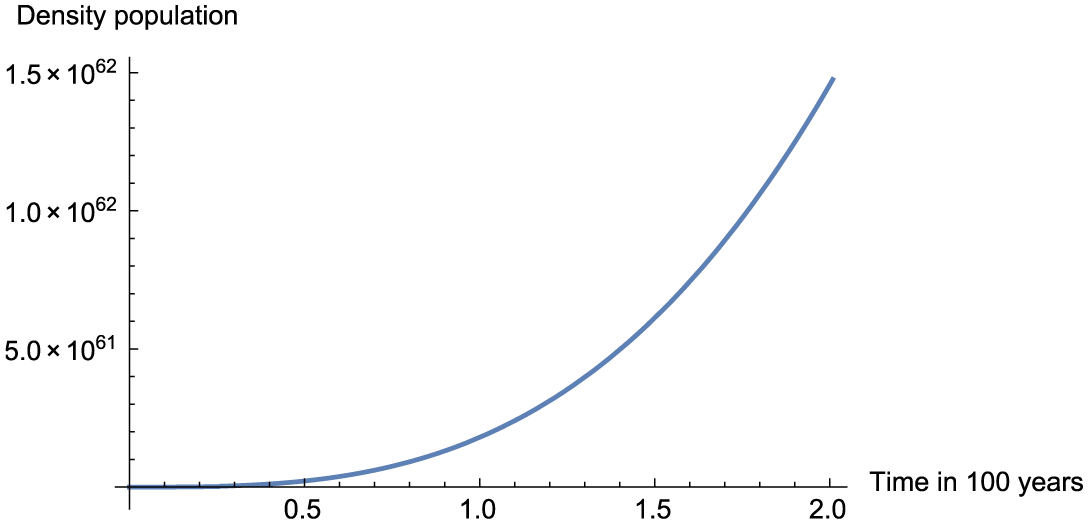
Numerical simulation for *α* = 0.75 and *t* = 100.

**Fig 3:**
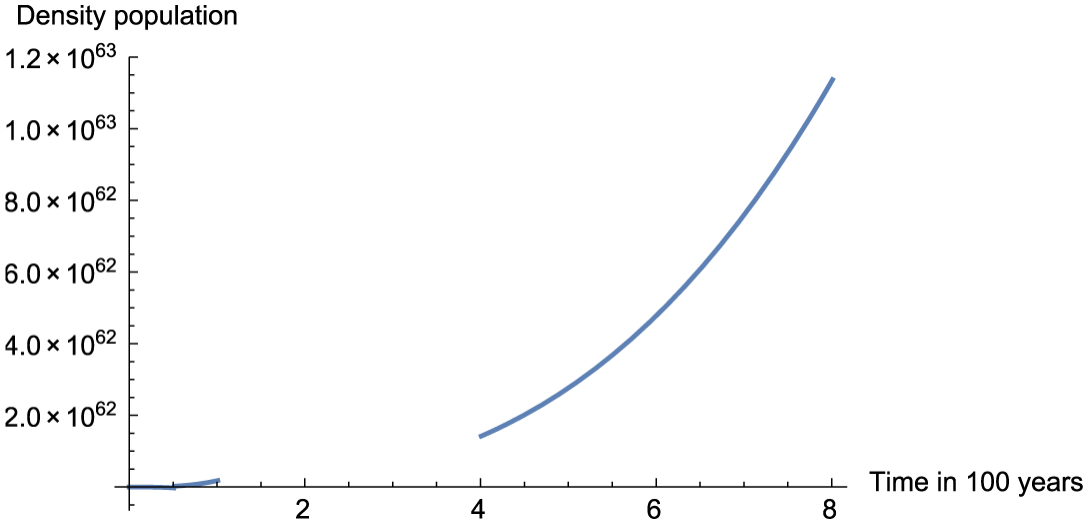
Numerical simulation for *α* = 0.45 and *t* = 100.

**Fig 4:**
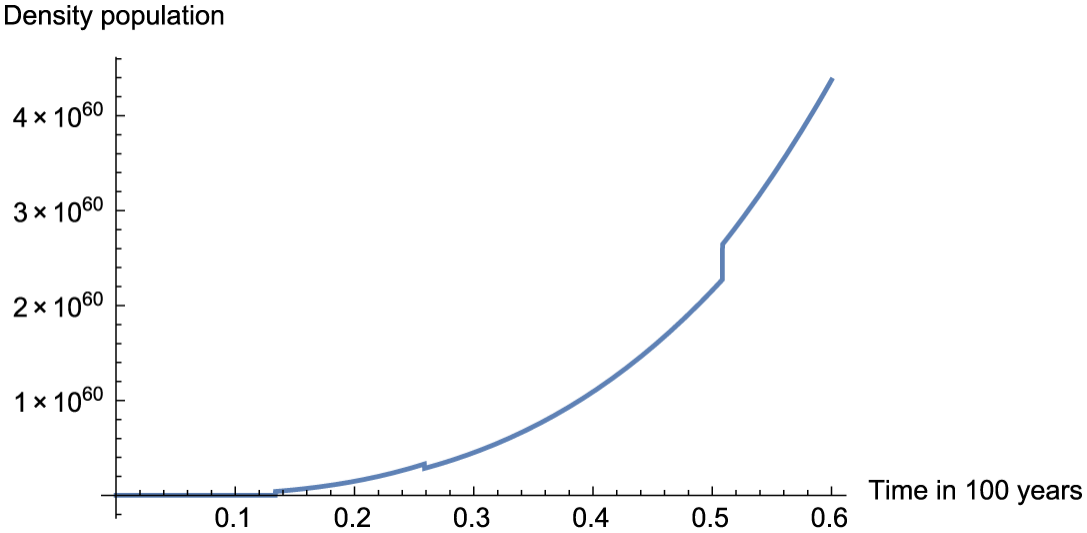
Numerical simulation for *α* = 0.25 and *t* = 100.

In this section, we present the numerical replication of the model for different values of fractional order using the proposed numerical scheme. The numerical solutions are depicted in Fig 1 for *α* = 0.95, Fig 2 for *α* = 0.75, Fig 3 for *α* = 0.45 and finally Fig 4 for *α* = 0.25.

## Conclusion

The aim of this work was to suggest a nonlinear fractional differential equation that could be used to describe the density of population growth taking into account real world behaviors. To do this, we introduced a new component that considers the choice of partner. The analysis of existence of positive solution of the new model was examined via the fixed-point theorem. The new model was solved numerically using the modified approach that fit well the new fractional integral. Some numerical simulations were done as function of fractional order.
